# Supplementary material for: Active site specificity profiling datasets of matrix metalloproteinases (MMPs) 1, 2, 3, 7, 8, 9, 12, 13 and 14
Source: Data Brief. 2016 Feb 22;7:299–310. doi: 10.1016/j.dib.2016.02.036 (PMC4777984; doi:10.1016/j.dib.2016.02.036)
Supplement: Supplementary file 10 — Supplementary material [file mmc10.zip › WebPICS_hMMP14_T_1%/P1.html]

 

PICS results


|  |  |
| --- | --- |
| **P1\_C**  7 in 307 sites   2.3 %    effects > 10 perc. pnts.  (vice-versa in brackets)  P1prime\_Q: 24.0 (12.0)   P3prime\_N: 23.4 (10.2) |  |
| **P1\_D**  20 in 307 sites   6.5 %    effects > 10 perc. pnts.  (vice-versa in brackets)  P3\_H: 12.4 (31.0)   P2\_E: 12.5 (10.9) |  |
| **P1\_F**  13 in 307 sites   4.2 %    effects > 10 perc. pnts.  (vice-versa in brackets)  P2\_M: 12.1 (15.8) |  |
| **P1\_G**  55 in 307 sites   17.9 %    effects > 10 perc. pnts.  (vice-versa in brackets)  P1prime\_W: 12.5 (57.1) |  |
| **P1\_M**  8 in 307 sites   2.6 %    effects > 10 perc. pnts.  (vice-versa in brackets)  P2prime\_W: 10.9 (17.4) |  |
| **P1\_N**  37 in 307 sites   12.1 %    effects > 10 perc. pnts.  (vice-versa in brackets)  P3prime\_L: 19.0 (21.2) |  |
| **P1\_P**  28 in 307 sites   9.1 %    effects > 10 perc. pnts.  (vice-versa in brackets)  P3prime\_S: 12.9 (14.0) |  |
